# Effect of interval and continuous small-sided games training on the bio-motor abilities of young soccer players: a comparative study

**DOI:** 10.1186/s13102-023-00664-w

**Published:** 2023-04-04

**Authors:** Farhad Daryanoosh, Hossein Alishavandi, Javad Nemati, Aref Basereh, Alireza Jowhari, Enayatollah Asad-manesh, Rafael Oliveira, João Paulo Brito, Pablo Prieto-González, Tomás García-Calvo, Kayvan Khoramipour, Hadi Nobari

**Affiliations:** 1 Exercise Physiology, Faculty of Educational Sciences and Psychology, Department of Sports Sciences, Shiraz University, Shiraz, Iran; 2 Department exercise physiology, Kharazmi University, Tehran, Iran; 3 Sports Science School of Rio Maior - Polytechnic Institute of Santarém, Rio Maior, 2040-413 Portugal; 4 Life Quality Research Centre, Rio Maior, 2040-413 Portugal; 5 Research Centre in Sports Sciences, Health Sciences and Human Development, Vila Real, 5001-801 Portugal; 6 Sport Sciences and Diagnostics Research Group, GSD-HPE Department, Prince Sultan University, Riyadh, 11586 Saudi Arabia; 7 Faculty of Sport Sciences, University of Extremadura, Cáceres, 10003 Spain; 8 Neuroscience Research Center, Institute of Neuropharmacology, Department of Physiology and Pharmacology, Afzalipour School of Medicine, Kerman University of Medical Sciences, Kerman, Iran

**Keywords:** Football, Body composition, Physical fitness, Aerobic power, Anaerobic power, Youth players

## Abstract

**Objective:**

The present study compared the effects of two different small-sided game (SSG) training methods, interval (ISSG) and continuous (CSSG) on the bio-motor abilities of young soccer players.

**Methods:**

Sixteen young soccer players (age: 19.5 ± 0.5 years; height: 177 ± 4.72 cm) were ranked based on the result of a running-based anaerobic sprint test (RAST) and randomly divided into two groups: CSSG (n = 8) and ISSG (n = 8). The training protocols were performed for eight weeks, three sessions per week. Participants were assessed twice (pre- and post-intervention) to estimate their anaerobic capacity with the RAST, aerobic capacity with Yo-Yo intermittent recovery test, body fat percentage with a bioimpedance analysis, speed with a 30-meter run test, and agility with the Illinois agility test. During the training session, the rating of the perceived exertion (RPE) and heart rate (mean and maximum) were recorded to assess the training load.

**Results:**

In general, aerobic and anaerobic capacities improved after ISSG (p < 0.05, for all). The between-group analysis with repeated measures ANOVA revealed higher values for ISSG than CSSG groups post-intervention in anaerobic power (p = 0.042, ηp^2^ = 0.264). In addition, the independent t-test results indicated that ISSG presented lower values of mean heart rate (p = 0.023, effect size [ES] = 0.85) and RPE (p < 0.05, ES = 0.88) than CSSG. Moreover, higher values for maximum heart rate were revealed for ISSG than for the CSSG group (p = 0.004, ES = 0.85).

**Conclusion:**

In conclusion, the findings of this study suggests that ISSG can lead to better improvements in anaerobic power and aerobic capacity than CSSG. Additionally, the ISSG led to a lower mean heart rate and RPE than the CSSG. Therefore, coaches and trainers may want to consider incorporating ISSG into their training programs for young soccer players to enhance their bio-motor abilities.

## Introduction

Small-sided Games (SSG) are training performed on smaller pitches, using modified rules with fewer players than traditional [1]. Currently, contradictory findings are available on how these exercises can optimally improve soccer players’ physical capacity and technical/tactical skills [2]. Nonetheless, a recent umbrella review of systematic reviews and meta-analyses about SSG reported several acute and chronic effects in tactical, technical, and physical dimensions, contributing to higher levels of physical fitness [3].

Several variables can affect the intensity of training during the SSG, such as the number of players; the pitch configuration (separate area by player and size of the field); if there is any specific rule for scoring a goal or not; using goalkeepers/jokers or not; the number of actions allowed; and the number of repetitions, sets, and ratio of effort/rest. The assumption that SSG simulates workloads, physiological loads, and intensities appropriate to the actual game and develops technical and tactical skills has led to their popularity among soccer coaches. Regular ball engagement may improve a soccer player’s performance more than any other method. SSG training can technically and physically create a match-like environment for players, recommending a maximum heart rate of 90 to 95% to improve and maintain cardiovascular fitness [4]. Previous research has shown that SSG can be as effective as traditional analytical methods of cardiorespiratory training for developing players’ physical fitness [5]. SSG offers advantages such as greater motivation, motor efficiency, tactical concentration, and technical ability in soccer athletes [6]. However, SSG’s effectiveness depends on how the drills are constrained, which can significantly impact physical and technical performance [7]. Additionally, SSG may have greater exercise intensity variability than traditional methods [8].

Some studies suggest specific exercises, such as soccer training in small areas, are preferred to improve aerobic capacity [9–11]. However, the contradictory research makes it impossible to draw provide a definite conclusion about SSG and how it affects physical fitness factors [12]. In a study by Hill-Hass et al. [13], it was reported that continuous SSG (CSSG) training has a higher fatigue index and heart rate than interval SSG (ISSG) training. Moreover, a previous systematic review [14] found that due to the variety of training designs and adaptations used in SSG, as well as the limited number of studies that have employed long-duration in SSG, it is not easy to draw a definitive conclusion about the impact of SSG on physical fitness factors. For instance, the previous systematic review [14] found only one study that used duration of 24 minutues [15]. Therefore, more research is needed to understand the effects of different types of SSG on physical fitness factors .

Therefore, the primary objective of this study is to compare the effects of ISSG and CSSG training with training durations between 25 to 40 minutes on the bio-motor abilities (anaerobic and aerobic capacities, body fat percentage, speed, and agility) of young soccer players. The study further seeks to understand the impact of the two training programs on the rating of perceived exertion (RPE) and heart rate (mean and maximum) of the participants during the training sessions to assess the training load and potential differences in intensity between the two programs.

The study hypothesis is that both ISSG and CSSG programs will enhance the bio-motor abilities of young soccer players. It is expected that both training programs will result in significant improvements in the physical fitness factors of the participants and that there will be no significant differences between the two programs in terms of the magnitude of the progress. Furthermore, the study will examine any differences in the training load and exercise intensity between the two programs, which could have implications for the design and implementation of training programs for young soccer players.

## Materials and methods

### Participants

First, a priori sample size calculation with G-Power software (University of Dusseldorf, Dusseldorf, Germany) with the following specifications was calculated: F tests through ANOVA with repeated measures, within-between interaction, effect size f = 0.30, α err prob = 0.05, power (1-β err prob) = 0.85, number of groups = 2, number of measurements = 6. The actual power output showed that 88.96% chance of successfully rejecting the null hypothesis of no difference in the variables in the study with 16 participants with sample size.

Thus, sixteen players, aged 19.5 ± 0.5 years, were randomly selected from 8 different national teams from the Iranian Youth League (3rd Division League, Omid) with a playing experience of 3–5 years. The eligibility criteria were as follows: (1) participants had no injuries, illness, or physical limitations during the study; (2) participants completed at least 80% of the total sessions; and (3) participants completed all of the test procedures (pre-post).

### Study design

This study followed a randomized parallel study design. After selecting the participants, all athletes were invited to Shiraz University, and a detailed explanation of the research procedure was provided. Then, they completed and signed the consent form. Before training, all participants were tested for Yo-Yo, Running-Based Anaerobic Sprint Test (RAST), Illinois agility, 30-meter speed, and body composition (body fat percentage). After that, the participants were ranked based on the result of the RAST test. Then they were divided into two groups (N = 8 in each group) of ISSG and CSSG using a matching design. Each group performed specific training protocols for 8 weeks in the pre-season period, 3 sessions per week (Table 1). After the end of 8 week-training protocols, the same tests were repeated. It should be noted that this study was conducted during pre-season when the players did not participate in any other training program rather than the training protocol of this study.

**Table 1:** The specific training protocols for 8 weeks for both groups

Time of CSSG	Time intervals of ISSG	Sessions
25 min	5 min, 1 min rest between intervals * 5	1–6
30 min	5 min, 1 min rest between intervals * 6	6–12
35 min	5 min, 1 min rest between intervals * 7	12–18
40 min	5 min, 1 min rest between intervals * 8	18–24

CSSG, continuous small-sided game; ISSG, interval small-sided game; min, minutes

For both CSSG and ISSG, the following characteristics were applied: field with 40x20 meters; four against four players; two turnovers were one point in favor of the opponent team; only two touches on the ball were allowed; spacing was encouraged by the researchers; the opposing team was trying to take the ball; each team that exchanged ten consecutive passes also scored one point. A team consisted of 2 midfielders, a forward, and a defender. Moreover, there was no predetermined intensity, and the players could do their best to win. The tests and training protocols were conducted on the artificial grass field of Shiraz University during the players’ pre-season from June to September 2018.

### Measurements

#### Heart rate

mean heart rate (HR_mean_) and HR maximal (HR_max_) were collected using a POLAR H10 (Polar Electro Oy, Kempele, Finland)participants’ heart rate were measured during each session.

#### Rate of perceived exertion (RPE)

using Borg [16] scale in all training sessions and each test. This scale is designed from 6 to 20. The number 6 indicates “very, very light”, and the number 20 indicates “very, very strong (almost maximal)”.

#### Anaerobic power

The RAST is a six by 35-m discontinuous sprint to measure anaerobic power[17]. Each sprint represents a maximal effort, with 10 s allowed between each sprint for turnaround. Two photocells measured the time for each run, and the start for each sprint (10-second interval) occurred with a beep from the photocell equipment. The athlete must sprint at maximum speed through the line each time. The next sprint starts from the opposite end of the measured track. The time between each run is designed to allow the athlete to return to the start line after running through the line to record the time and reset the watch. At the end of the test, the coach will have six times which can be used, along with body weight, to calculate maximal, minimal and average power outputs along with a fatigue index as follow [18, 19].

Power = Body Mass × Distance ² ÷ Time ³

Maximum power - the highest value

Minimum power - the lowest value

Average power - the sum of all six values ÷ 6

Fatigue Index - (Maximum power - Minimum power) ÷ Total time for the 6 sprints

For calculating maximum power, the lowest time, minimum power, the highest time and mean power, the mean of 6 intervals were used.

#### Aerobic power

The Yo-Yo intermittent recovery test level 1 consists of repeated 2 × 20-m runs back and forth between the starting, turning, and finishing line at a progressively increased speed controlled by audio bleeps from a tape recorder and it was used to measure anaerobic power. The subjects have a 10-s active rest period between each running bout, consisting of 2 × 5-m jogging. When the subjects twice failed to reach the finishing line in time, the distance covered is recorded and represents the test result. The test consisted of 4 running bouts at 10–13 km·h^-1^ (0 − 160-m) and another 7 runs at 13.5–14 km·h^-1^ (160–440-m), where after it continues with stepwise 0.5 km·h^-1^ speed increments after every 8 running bouts (i.e., after 760, 1080, 1400, 1720-m, etc.) until exhaustion [20, 21]. The test was performed indoors on running lanes, marked by cones, having a width of 2-m and a length of 20-m. Another cone placed 5-m behind the finishing line marked the running distance during the active recovery period. Before the test, all subjects carried out a warm-up period consisting of the first four running bouts in the test [22].

After the test, the maximum oxygen consumption (VO_2max_) was calculated using the following formula [23]:

VO_2max_ (ml·min^-1^·kg^-1^) = distance run × 0.0136 + 45.3

#### Speed

A 30-meter speed test measured the players’ speed. Each subject ran the 30-meter route thrice at a maximum speed, and the best time of each player was recorded [24, 25].

#### Agility

The Illinois agility test was used to measure agility. Participants travelled at a maximum speed similar to the route drawn, and the time was recorded [26]. The length of the course was 10-m, and the width was 5-m. Four cones were used to mark the start, finish and the two turning points. Another four cones were placed down the centre an equal distance apart. Each cone in the centre was spaced 3.3-m apart. The player lies in the prone position with his chin touching the surface of the starting line. The first light sensor is placed 50-cm above the ground at the start line and it was activated when participants move from the prone position. The second light sensor was placed at the finish line Timing gates were placed at the start and finish lines at a height of 30-cm. On the researchers “Go” command, the stopwatch was started, and the participant got up as quickly as possible and ran around the course in the direction indicated while attempting to avoid contact with the placed cones. He then runs towards the starting line’s middle cone, zig-zags through the cones downward and again upwards, sprints to the last cone on the far side and finishes at the finish line. Upon crossing the finish line, the timing was stopped. Subjects performed two maximal attempts at each exercise with at least 2 min rest between tests and trials. The faster time taken and recorded in seconds [27]. This test was valid and reliable in male team sports athletes [28].

#### Body composition

participants’ body weight and fat percentage were evaluated using a body analysis device (MA601, Taichung City 41,262 Taiwan) [29].

#### Diet

participants’ diet was controlled using food processor nutrition analysis software (PCN software; Cesnid, Spain).

### Statistical analysis

Mean, and standard deviation (SD) were used to describe data. Then, the normality and homogeneity of the data were assessed using Shapiro-Wilk and Levene tests, respectively. Repeated measures ANOVA (2×2) test was used to evaluate the Aerobic and Anaerobic power, Agility, Speed, Body fat Percentage and Body mass variables in both ISSG and CSSG groups. In addition, an independent t-test was used to compare between groups’ differences in RPE, mean, and maximum heart rate. A p < 0.05 was considered significant and partial eta squared was used as effect size where the following thresholds were applied: ηp^2^ = 0.01 indicates a small effect. ηp^2^ = 0.06 shows a medium effect. ηp^2^ = 0.14 indicates a large effect [30].

## Results

### Aerobic power

At baseline, there were no differences in aerobic power (VO_2max_) between groups (p > 0.05). The repeated measures ANOVA results related to aerobic power showed no significant differences for a GROUP×TIME interaction (F(1,14) = 1.65, p = 0.219, ηp^2^ = 0.588).

### Anaerobic power

**Fig. 1:**
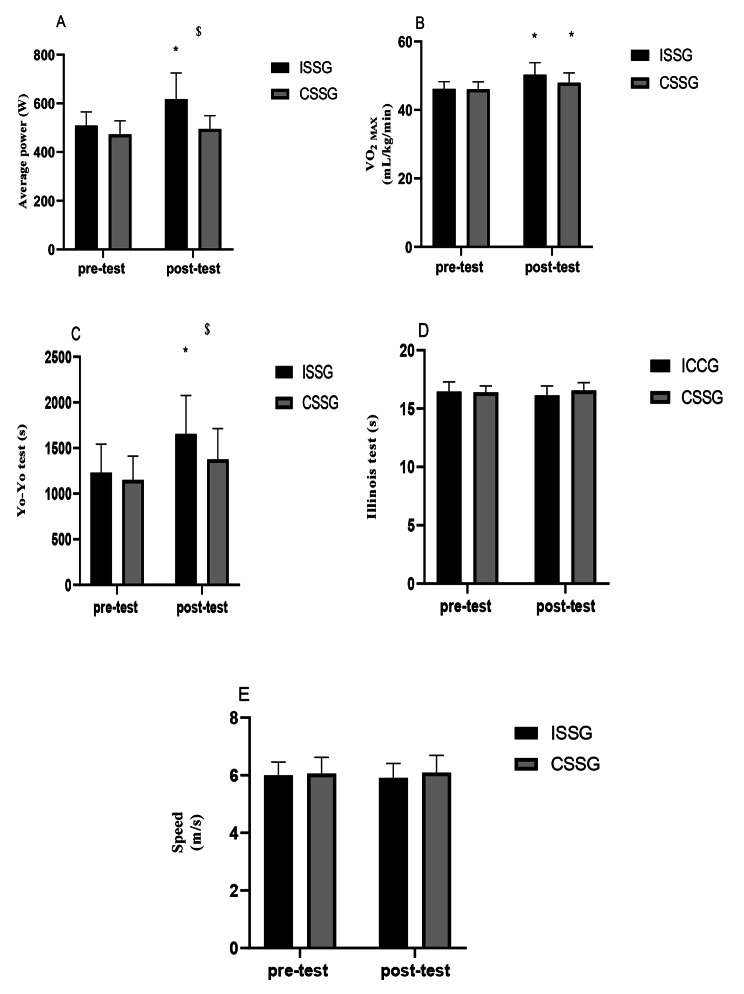
The pre-test and post-test (A) Average power, (B) Aerobic power, (C) Yo-Yo Test, (D) Illinois agility and (E) 30-m test in both groups. The sign $ indicates a significant difference between the two groups and the sign * indicates a significant difference between pre to post training

At baseline, there were no differences in anaerobic power between groups (p > 0.05). The results of the repeated measures ANOVA showed that there was a significant difference in main effect of time (F(1,14) = 11.29, p = 0.005, ηp^2^ = 0.446) and a GROUP×TIME interaction (F(1,14) = 5.01, p = 0.042, ηp^2^ = 0.264). The interaction results showed that the ISSG group significantly increased anaerobic capacity more considerably than the CSSG group (p = 0.005) (Fig. 1).

### Yo-Yo test

At baseline, there were no differences in the Yo-Yo test between groups (p > 0.05). The repeated measures ANOVA test results showed no significant difference for a GROUP×TIME interaction (F(1,14) = 1.93, p = 0.186, ηp^2^ = 0.121). (Fig. 1).

### Agility

At baseline, there were no differences between groups in the Illinois agility test (p > 0.05). The results of the repeated measures ANOVA test related to the Illinois agility test showed that there was no significant difference for TIME (F(1,14) = 0.332, p = 0.573, ηp^2^ = 0.023) and a GROUP×TIME interaction (F(1,14) = 2.69, p = 0.123, ηp^2^ = 0.161) (Fig. 1).

### Speed

At baseline, there were no differences in the 30-m test between groups (p > 0.05). The results of the repeated measures ANOVA test showed that there was no significant difference for TIME (F(1,14) = 0.316, p = 0.583, ηp^2^ = 0.022) and a GROUP×TIME interaction (F(1,14) = 1.40, p = 0.255, ηp^2^ = 0.091) (Fig. 1).

### Body fat Percentage

**Fig. 2:**
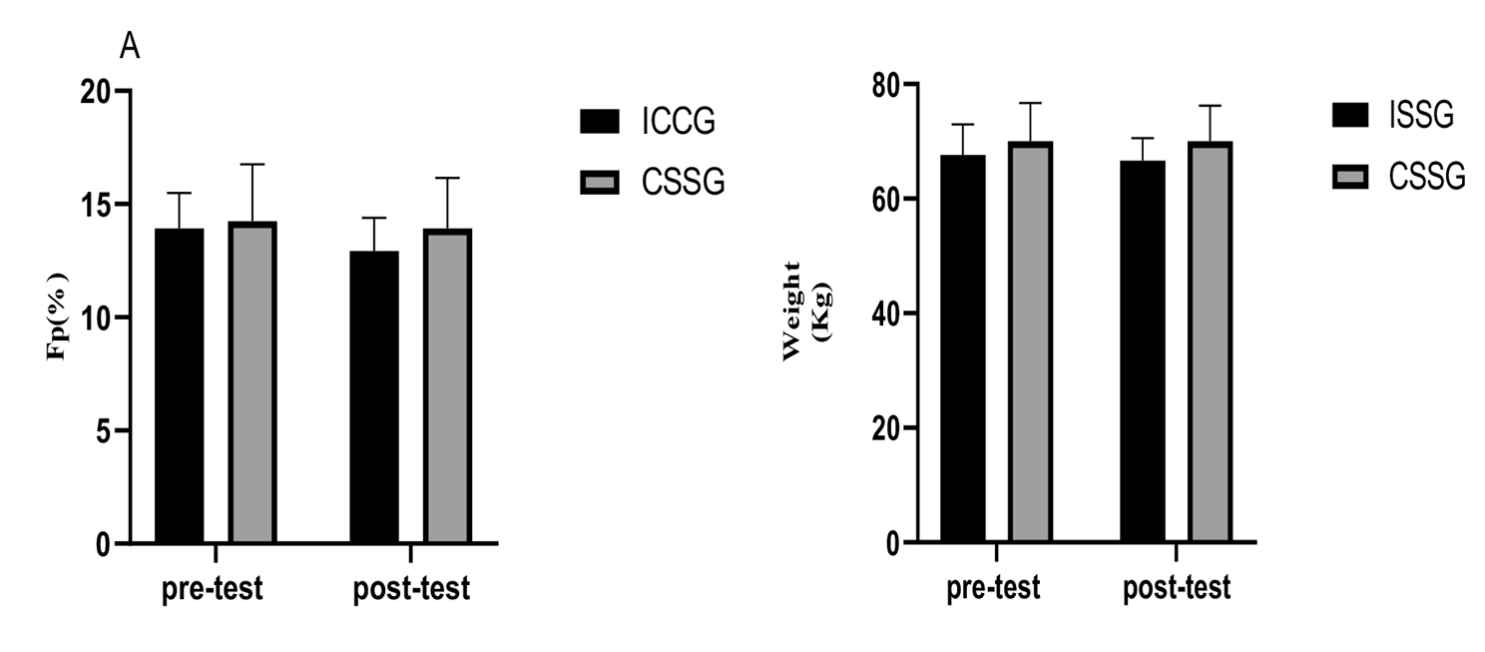
The pre-test and post-test (A) Body fat percentage changes and (B) Body weight measured in both groups

At baseline, there were no differences in body fat percentage between groups (p > 0.05). The results of the repeated measures ANOVA test showed that there is no significant difference for TIME (F(1,14) = 4.01, p = 0.065, ηp^2^ = 0.223) and a GROUP×TIME interaction (F(1,14) = 1.10, p = 0.312, ηp^2^ = 0.073) (Fig. 2).

### Body mass

At baseline, there were no differences in Body mass between groups (p > 0.05). The results of the repeated measures ANOVA test showed that there was no significant difference neither for Time (F(1,14) = 0.903, p = 0.358, ηp^2^ = 0.061) nor for GROUP×TIME interaction (F(1,14) = 0.902, p = 0.358, ηp^2^ = 0.060) (Fig. 2).

### Mean heart rate

**Fig. 3:**
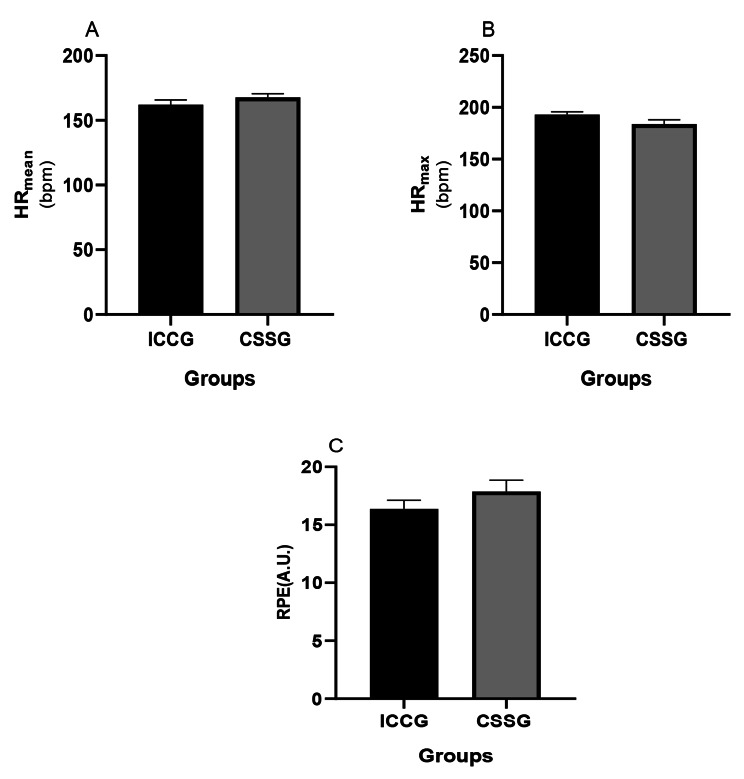
Mean and standard deviation of changes in (A) mean heart rate, (B) the maximum heart rate and (C) RPE in both groups. The sign $ indicates a significant difference between the two groups

The mean and SD of HR_mean_ were 162 ± 3.67 in the ISSG group and 168 ± 2.86 beats per minute (bpm) in the CSSG group. The HR_mean_ in the CSSG group was significantly higher than the ISSG group (t8 = 2.74, p = 0.023, ES = 0.85) (Fig. 3).

### Maximum heart rate

The mean and SD of the HR_max_ changes were 193 ± 2.82 in the ISSG group and 184 ± 4.32 bpm in the CSSG group. The HR_max_ in the ISSG group was significantly higher than the CSSG group (t8 = 3.98, p = 0.004, ES = 0.85) (Fig. 3).

### RPE

The mean and SD of RPE were 16.37 ± 0.74A.U. In the ISSG group and 17.87 ± 0.99A.U. In the CSSG group. RPE was significantly higher in the CSSG group (p = 0.024, ES = 0.88) (Fig. 3).

## Discussion

This study aimed to compare the effect of ISSG and CSSG on the bio-motor abilities of young soccer players. Our results showed that aerobic power (i.e., VO_2max_ and Yo-Yo), and anaerobic power (RAST) improved in both groups. In contrast, the results of Illinois, 30-meter, body fat percentage, and body weight showed no improvements. Finally, the intensity measures of HR_mean,_ and RPE were lower in ISSG than CSSG, while HR_max_ was higher in ISSG than in CSSG groups.

### Aerobic power

In the present study, it was found that there is no significant difference between the two groups of ISSG and CSSG in VO_2max_ and Yo-Yo test results. However, after comparing the pre-test and post-test in both, it was found that there was a significant increase only in the ISSG training group. Previous research was consistent with the results of the present study [31]. That study compared traditional (running) with SSG training, in which SSG training was performed in the form of intervals in 4 sets of 4 minutes with a maximum heart rate of 90 to 95% and 3 minutes of recovery between the sets. The results of that study showed that the sub-maximal lactate response and VO_2max_ were improved in those players. Also, with the increase in aerobic abilities of the players, their displacement in the game increased by 571 meters [31]. Another study compared ISSG and CSSG (2 vs 2, 3 vs 3 and 4 vs 4) training with 6–12 minutes of duration and found similar physiological responses in both groups, including improvement in VO_2max_ [32]. Another study found that SSG (5 vs 5) training performed with 85% HR improved aerobic power [33]. However, other research found that SSG training did not affect participants’ aerobic capacity compared to non-ball speed training. Still, it is important to highlight that floaters were used and could justify the different results [34]. Besides, the number of players and the field size could also justify the different results from the present study.

### Anaerobic capacity, heart rate, maximum heart rate and RPE

The present study found that anaerobic power in the ISSG training group was higher than in the CSSG group. Also, after comparing the pre-test and post-test in both groups, it was found that there was a significant increase only in the ISSG training group. On the other hand, the HR_mean_ in the CSSG group was significantly higher than in the ISSG training. Also, there was a significant increase in the HR_max_ in ISSG compared to CSSG. CSSG allowed players to sustain a higher work rate over time. Despite the higher training volume, the locomotor demands tend to be low, producing lower fatigue [35]. CSSG seems to allow a better recovery for a strength training day, contributing to better readiness in the following days [36]. Also, this training type can contribute to tactical complexity improvements [5].

In the present study, heart rate (HR) was used to evaluate the effect of CSSG and ISSG training on the physical fitness of young soccer players. CSSG training produced a higher HR response than ISSG training, consistent with previous studies using a similar training design [14]. The consistent intensity of CSSG training is the main contributor to this effect. On the other hand, ISSG training is characterized by constantly varying exercise intensity over a short period, causing HR to peak and then decline. This exercise pattern resulted in a significant increase in mean HR of the ISSG group compared to the CSSG group. In addition, the ratings of perceived exertion (RPE) of the CSSG group were significantly higher than those of the ISSG group, indicating that the CSSG exercise was more intense. Further research could explore these different training methods’ potential advantages and disadvantages in more detail.

In line with the results of the present study, we can refer to a study where the authors compared the two types of ISSG training (3 6-minute attempts with 3 minutes recovery between) and CSSG (an 18-minute effort without recovery). The authors concluded similar improvements for the anaerobic power index, while HR, RPE and blood lactate were identical in both groups [37]. In another study, the effects of CSSG (2 vs 2, 3 vs 3, and 4 vs 4 with a duration of 6, 9, and 12 minutes, respectively) and ISSG (2 vs 2, 3 vs 3, and 4 vs 4 with a duration of 2, 3, and 6 minutes, respectively) were compared. The results indicated a significant improvement in anaerobic power for all three protocols [32]. In another study, the ISSG (4 vs 4 with 3 of 6-minute attempts with varying recovery times between at-tempts) observed a significant improvement in anaerobic power in the post-test compared to the pre-test [38].

The literature shows subtle differences in training programs, age, and player ability. Based on the research background, the simultaneous increase in the number of players and the size of the field in SSG increases the training intensity. For example, Rampini et al. examined the effects of a simultaneous increase in the number of players and pitch on HR_max_, blood lactate and RPE in 20 amateur soccer players. The results showed that an increase would follow the training intensity in the named variables [31]. On the other hand, they found that when the number of players increased, they reported a decrease in the HR_max_ percentage. The heart rate increased as the number of players decreased [12]. In contrast, other studies did not show significant results in HR changes with decreased players [32, 39, 40].

Most research suggests that as the field size increases, RPE, HR, and lactate concentration increase [12, 41, 42]. A study of the effect of ground dimensions on HR showed that with increasing ground dimensions, heart rate increased during activity [31, 43]. In contrast, a previous study has yet to achieve significant results [12]. Two studies also observed higher RPE with increasing field dimensions [31, 43].

Recent studies have shown that different numbers of players obtain different physiological and technical responses. With the number of players decreasing, heart rate, fatigue index and lactate concentration increase, but technical activities decrease. The relationship between the parameters of small-sided game training and the players’ ratio to the field’s size is also important [36].

### Body mass and fat, speed and agility

In the present study, the changes in soccer players’ body mass and fat percentage decreased in both training programs, but this decrease was not significant. It seems that the reason for the non-significance of these changes goes back to the initial body weight and the level of physical fitness of these players, and also the insufficient training period (eight weeks), which is in line with previous a previous study that analysed the effect after a detraining period of four weeks plus a training period of another four weeks [44]. Indeed, another study showed positive effects only after 11 weeks [45]. Also, in the variables of speed and agility, the results showed that the speed was not significantly different between the two groups of ISSG and CSSG. An increase in speed was observed in both groups, which was greater in the ISSG training group; however, none of the results related to this variable was significant. In the analysis of agility, no significant difference was observed between pre-test and post-test values for each group. Among the reasons why speed and agility in soccer players were not significant in this study could be related to the short training period and the players’ initial level of physical fitness.

The present study has some limitations that could affect the interpretation of the results. One limitation is the small sample size, which may not represent the young soccer players’ population. This limitation may affect the external validity of the study results, as the findings may not be generalizable to other people or contexts. Nonetheless, the sample size calculation showed 88.96% of power with 16 participants. Additionally, only 4vs4 SSG were considered for analysis, which may limit the generalizability of the results to other SSG formats. Furthermore, the study only analyzed a short period of eight weeks of the pre-season, which may not capture the long-term effects of the different SSG interventions. Finally, the lack of control over confounding variables, such as dietary habits or other physical activity outside the training program could be considered another limitation. These factors could potentially bias the results of the study in either direction.

Despite these limitations, the present study has practical implications for coaches and trainers who work with young soccer players. The findings suggest that both ISSG and CSSG can effectively enhance the bio-motor abilities of young soccer players, but with different physiological responses. Coaches and trainers can use this information to tailor their training programs to their athletes’ specific needs and goals. Future research should address the present study’s limitations by using larger sample sizes, longer intervention periods, and more comprehensive measures of physical activity and dietary habits. Additionally, future studies could investigate the effects of SSG interventions on other outcomes, such as technical and tactical performance, injury risk, and psychological factors. The present study provides a foundation for further research on SSG interventions in young soccer players.

Overall, the results of this study help coaches and their staff to optimize their training plan and periodization by providing highlights with CSSG and ISSG with total durations between 25 to 40 minutes. Additionally, this study provides relevant information on two possibilities of SSG in different formats (intermittent and continuous), which can be chosen according to the training’s aim and objectives and the period of the season.

## Conclusion

The present study shows that ISSG training has a greater effect on improving anaerobic power, but CSSG also improved, although with a lower magnitude. In addition, according to the obtained RPE and HR results, the degree of difficulty of ISSG training is lower than CSSG training.

## Data Availability

The datasets generated during and analyzed during the current study are available from the author F.D and KK. upon reasonable request.
